# Numerical modelling of soldered superconducting REBCO stacks of tapes suggests strong reduction in cross-field demagnetization

**DOI:** 10.1038/s41598-023-27996-4

**Published:** 2023-01-19

**Authors:** Shuo Li, Enric Pardo

**Affiliations:** 1 Institute of Electrical Engineering, Slovak Academy of Sciences, Bratislava, Slovakia; 2 College of Information Science and Engineering, Northeastern University, Shenyang, 110819 China

**Keywords:** Electrical and electronic engineering, Applied physics

## Abstract

Stacks of superconducting REBCO tapes (or “stacks”) can trap high magnetic fields, above 17 T. However, relatively low oscillating transverse magnetic fields can fully demagnetize the stacks. This is an issue if the stacks act as poles in the rotor of a superconducting motor, for instance. Here, we study the drastic suppression of cross-field demagnetization in stacks by soldering the tapes at the ends using a normal conductor. In particular, we analyzed by numerical modeling a stack of five REBCO thin films connected at the ends by resistances. The computed trapped field of a stack with zero solder resistance decays very fast at the beginning but then tends to stabilize to relatively high values, while the trapped field of an isolated stack (infinite resistance at the ends) decays further (it decays to zero if the transverse field is above the parallel penetration field). For intermediate solder resistances, the stable value of the trapped magnetic field is in between those of the isolated and zero-resistance configurations. Since the stable trapped field in soldered stacks increases with the number of tapes, stacks of sufficiently high number of soldered tapes could be immune to cross-field demagnetization. This opens the gate for a new kind of superconductors that mostly behave as bulks, especially if the stacks are made of delaminated tapes or it is possible to solder the tapes by very low resistance.

## Introduction

Superconducting bulk acting as trapped field magnets can achieve a magnetic field magnitude over ten times greater than the maximum field of conventional permanent magnets. Until now, the world record by an MgB$_2$ cylinder bulk is 16.1 T^1^ and by a REBCO superconductor bulk is 17.6 T^2^ compared to the saturation magnetization 1.3–1.4 T for typical NdFeB. Compared with superconducting bulk, a superconducting stack has many advantages. The stack width can be over 40 mm^3^ and larger continuous superconductor shapes and result in larger trapped flux for the same maximal trapped field.

Accordingly, the stack can trap a higher magnetic field than the bulk, setting the world record of 17.7 T^4^, where the limitations are mechanical issues. The HTS stack is attractive for a wide range of applications, such as superconducting motors^5,6^, magnetic bearings^7^ and so on.

The reason is that superconducting stacks in the rotor of motors can trap larger magnetic fields with no need for current leads, compared to the field winding^8^ or cages^9^ that increase the device complexity. Therefore, HTS stacks have a high potential for electric aircraft because they can reduce weight and gain high power density^10–14^.

As reported in^10–14^, the high power density superconducting motor designed for electric aircraft propulsion adopts the superconducting stacks as magnet poles in the rotor. The stacks should first be properly magnetized and then try to keep the field for hours to ensure that the motor can operate at the rated level throughout the flight. When the motor is operating, in the air gap of an electric motor there are always excessive harmonics caused by the differences in permeability of the magnetic circuit (slot harmonics) and the discrete distribution of the stator coils (winding harmonics). It has been reported from laboratory testing and computer modeling that the cross-field is of the same order of magnitude as the parallel penetration field in each layer, which could demagnetize the stack in just a few minutes^15,16^. The cross-field that the stacks experience in the rotor causes demagnetization of the trapped field, which reduces the performance of the motor^17,18^. This decay must be carefully considered when designing the stacks for motors.

There has been agreat effort to fully understand cross-field demagnetization to reduce the demagnetization effects and extend the time of the trapped field inside the stack. Patel and Baghdadi et al. reported the experiment method and results about a stack of REBCO tapes after 100 cycles for applied crossed field amplitude in the range of 800–300 mT and frequency 0.5 and 2.5 Hz^19,20^. Later, they extended the cross-field to 30000 cycles and frequency ranging from 1 to 100 Hz^21^. Campbell et al. studied the demagnetization of the cross-fields in the superconductor stack numerically and experimentally and presented the current distribution in the superconductor stack with 8 tapes^15^. Li et al. measured and calculated the decay of the trapped magnetic field in a stack subjected to traveling magnetic waves with the H-formulation method^22^. Tomkow and Smara et al. studied the distribution of the trapped magnetic flux in a superconducting stack magnetized by an angled field^23^. Smara then reported an experimental assessment of superconducting stack demagnetization in which stacks are used as magnetic poles in the rotor^11^. In motors for aviation, the base frequency of the stator is of few hundreds of Hz^24^. Actually, the magnetic field in the rotor stacks presents also higher harmonics due to the winding shape and iron poles, if present. The resulting harmonics can be of up to few kHz.

It is technically possible to make isolated stacks, which are already used in the ASuMED project^24^. The motivation for studying soldered stacks is that bulks with the same engineering current density are much less sensitive to cross-field demagnetization, as shown in^16^. We expect that soldered stacks will behave as bulk for large enough frequencies of the ripple field. When the tapes are soldered together, there is resistance between the tapes. The resistances will affect the trapped field and the current front in tapes when the stacks suffer from cross-field. Although several publications have appeared addressing the question of the demagnetization of the stacks in laboratory conditions, and some solutions are even described in^25^, there seems to be a lack of sufficient data on how a superconducting stack with resistances will behave in the air gap of an electric machine. So, in this paper, we will systematically study the trapped field demagnetization of a partially coupled stack with consideration of resistance between tapes under a time-varying alternating cross-field by minimum electromagnetic entropy production 2D method (MEMEP 2D). Soldered self-supporting stacks have been already experimentally constructed in^26,27^, which did not experience mechanical issues. However, cross-field demagnetization on these stacks has not been studied.

Right now, commercial finite element methods with the H-formulation method and the T-A formulation are very popular and flexible in superconducting modeling^13,28–30^. However, it takes too much computing resource and time, which is not suitable to model the trapped field of the partially coupled stack with resistance at terminals suffering from thousands of cycles cross-field, because the tapes need to be meshed into many elements in thickness to get sufficient precision result, which causes unfeasibly high computation times.

The MEMEP 2D is much faster than the H-formulation and T-A formulation methods, so it can adapt a much better mesh strategy to get high precision results^31–34^. Pardo and Kapolka et al. presented a 3D modeling of the magnetization of superconducting rectangular and cubic bulk and stack based on the MEMEP method^35–37^. Then, Pardo and Dadhich et al. systematically studied cross-field demagnetization of stacks and the time constant of the transverse field demagnetization of superconducting stacks with MEMEP 2D and 3D methods^38,39^. Li and Pardo presented a modified MEMEP 2D model to calculate the partially coupled stack with consideration of the resistances between tapes which show high accuracy and are verified by measurement^40^. With the help of the model, Li then studied the resistance dependence on the magnetization loss for a partially coupled stack and quasi-isotropic strands in^41–43^. Thanks to the numerical MEMEP 2D model, we can also study the cross-field demagnetization of the partially coupled stack much easier and faster.

The structure of the paper is as follows. The general minimum electromagnetic entropy production 2D method for a stack of REBCO tapes with resistances between tapes at terminals is presented briefly in Section “Numerical modeling”. Then, the superconducting stack configuration is described in Section “Modeling configuration”. Later, the results of the trapped field demagnetization for the partially coupled stack over eight thousand cycles of time-varying cross-field are presented and discussed in Section “Results and discussion”. At last, conclusions are given in Section “Conclusion”. Besides, the results of the magnetization loss under the cross-field are presented in the supplementary material.

## Numerical modeling

Usually, the HTS stack can be classified as uncoupled stack (insulated between tapes, $R_{av} = \infty$ $\Omega$) , fully coupled stack (connected with zero resistance,$R_{av} = 0$ $\Omega$), and partially coupled stack (joined by a finite resistance, $0<R_{av} < \infty$ $\Omega$). For the partially coupled stacks, the superconductor tapes are connected at terminals with conventional materials, and the tape-to-tape currents are induced when a parallel time-varying alternating magnetic field is applied, which causes additional resistance loss at the same time. The induced currents appear not only in superconductors, but also in the resistances between tapes. In order to take tape-to-tape currents into account, we developed a numerical model based on the MEMEP 2D method in our previous work^40^ (see more details in 6). This model can calculate the current density in superconductor tapes. The modified MEMEP 2D in^40^ takes normal connections at the tape ends into account. Here, we assume that the tapes are connected at the ends only, which enables 2D modeling. Continuous connection along the length will require computationally extensive 3D modeling.

Once the current density is calculated, we can deduce the trapped field function above the stack at each time step by Biot-Savart’s law as follows,

1 $$B_{trapX}(t)= \frac{2\pi }{\mu _{0}} \sum _{1}^N \frac{(y_i -y_p)\times I_i(t)}{(x_i-x_p)^2+(y_i-y_p)^2}$$

2 $$\begin{aligned} B_{trapY}(t)= & {} \frac{2\pi }{\mu _{0}} \sum _{1}^N \frac{(x_p -x_i)\times I_i(t) }{(x_i-x_p)^2+(y_i-y_p)^2} \end{aligned}$$

where $\mu _{0}$ is permeability of vacuum ($4\pi \times 10^{-7}$ H/m). ($x_p$ , $y_p$ ) are the observation point coordinates , $(x_i,y_i)$ are the coordinates of the center of element *i* in the superconductor, $I_i(t)$ is its current at time *t*, $B_{trapX}$ and $B_{trapY}$ are the trapped field of the stack in *x* and *y* directions respectively. In this article, $B_{trapY}$ is also referred as $B_{trapped}$.

## Modeling configuration

**Figure 1:**
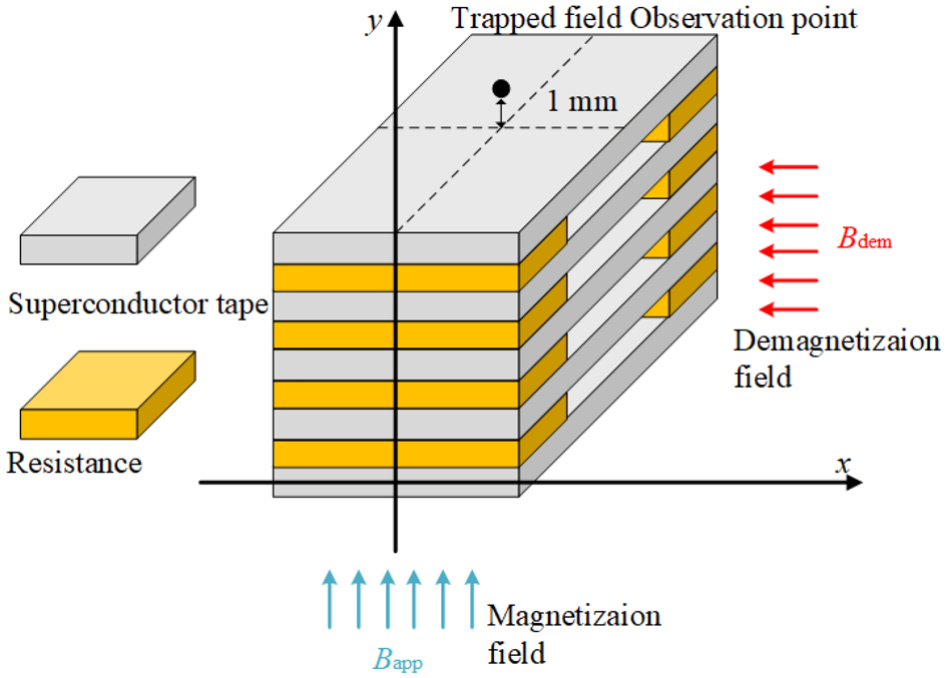
A sketch of the superconducting stack with resistances at terminals. The gray parts are the superconducting layers and the yellow parts are the resistances between tapes. A field along *y* axis is applied ($B_{app}$) on the HTS tapes to magnetization. Then, an alternating triangular magnetic field along the *x* axis is applied ($B_{dem}$) to demagnetize the stack. The trapped magnetic field $B_{trapped}$ (along *y* axis direction) is observed at 1 mm above the top tape surface center.

A partially coupled superconducting stack with 5 tapes and resistances at terminals is modeled. The superconductor tape is 40 mm in width and 18 cm in length, with assumed critical current of 3702.4 A at 25 K. These parameters are relevant for RABiTS-based REBCO tapes, such as those from American Superconductor (AMSC). Indeed, this critical current or higher could be achieved with AMSC (coil formulation) at 25 K and magnetic fields below 1 T (see dataset in^44^ of the database in^45^ obtained as detailed in^46^). We chose this tape width, intead of 12 mm offered from other producers, since wider tapes are more interesting for applications due to their superior trapped field. Because mainly the superconducting layer can trap the magnetic field and other layers (such as the metallic substrate layer and silver thin layer) can only induce negligible eddy current losses, the model only takes the superconducting layer into account. The resistances between tapes are taken into account precisely because the resistances can induce tape-to-tape current under parallel cross alternating magnetic field and then affect the current front in tapes, sequentially on the trapped magnetic field. Detailed parameters of the stack are provided in Table 1 and a sketch of the partially coupled stack is illustrated in Fig. 1.

**Table 1:** The specific parameters of the superconducting stacks used for the calculations.

Parameters	Description	Value
$I_c$	Self-field critical current	3702.4 [A]
*n*	*n*-value	30
$\mathrm T$	Working temperature	30 [K]
$w_{s}$	Width of the superconducting layer	40 [mm]
$h_{s}$	Thickness of the superconducting layer	1.5 [μm]
$n_{tape}$	Number of tapes in the stack	5
$d_{tape}$	Distance between the tapes	1.5 [mm]
$l_{s}$	Length of the stacks	180 [mm]
$R_{av}$	Average resistance at each end	0.05, 0.5, 5, 50 $[\upmu \Omega ]$
$B_{m}$	Amplitude of the applied alternating cross field	100, 200, 500 [mT]
*f*	Frequency	2400 [Hz]

The assumed critical current is lower or equal to extrapolated results in^44^ at 25 K and magnetic fields below 1 T.

We chose a tape-to-tape separation of 1.5 μm in order to achieve a total stack thickness (6 mm) of the same order of magnitude as stacks to be used in rotors of rotating machines^5,24,47^, but with only 5 tapes for computational feasibility. Although the stacks will use low tape-to-tape separation and high number of tapes, the average electromotive force due to the ripple field will be the same. Thus, we expect the same qualitative (and maybe even quantitative) behavior as for our simpler 5-tape stack, but the stationary state in actual stacks will likely require higher number of cycles due to the inductive effects caused by the higher number of tapes^16,39^.

This configuration could be directly compared to experiments of tapes soldered at the ends. In order to compare to tapes soldered along the whole length instead of only at the ends, we can approximate that the resistance between tapes, $R_{av}$ , corresponds to assuming that tape is soldered on one fourth of the tape length close to each end (Fig. 1). Then, $R_{av}=4R_s/S$ , where $R_s$ is the surface resistance between tapes and *S* is the total surface soldered between tapes. In our configuration, $R_{av}=aR_s$ with $a=555.6$  m$^{-2}$ . Previous experiments at 77 K showed that it is possible to simply solder tapes face-to-face with surface resistances as low as^48^ $R_s=380$ n$\Omega$ cm$^2$ = 3.8$\cdot 10^{-11}$ $\Omega$ m$^2$ , which results in $R_{av}$ = 2.111$\cdot 10^{-8}$ $\Omega$ . Experiments in^40^ show resistance between copper-stabilized tapes soldered face-to-back with resistance $R_{av}'=3.13\ \upmu \Omega$. Assumming the pessimistic scenario that the tape-to-tape resistance is dominated by the copper stabilization on the edges and that in^40^ the length of the solder is $l'=5$ mm, the resistance between tapes for our case will be $R_{av}=R_{av}'l'/l$, resulting in $R_{av}=3.5\cdot 10^{-7}\ \Omega$. If the wide tapes are not copper stabilized (only thin silver stabilization), we can expect higher tape-to-tape resistances. For that reason, we decided to study $R_{av}$ values ranging from $5\cdot 10^{-8}$ to $5\cdot 10^{-5}\ \Omega$. Besides, our results below show that for $R_{av}<5\cdot 10^{-8}\ \Omega$ the decay of the trapped field is almost the same as for $5\cdot 10^{-8}\ \Omega$ and for $R_{av}>5\cdot 10^{-5}\ \Omega$ the trapped field is close to that for $5\cdot 10^{-5}\ \Omega$. Then, this range covers all possible qualitative behavior.

**Figure 2:**
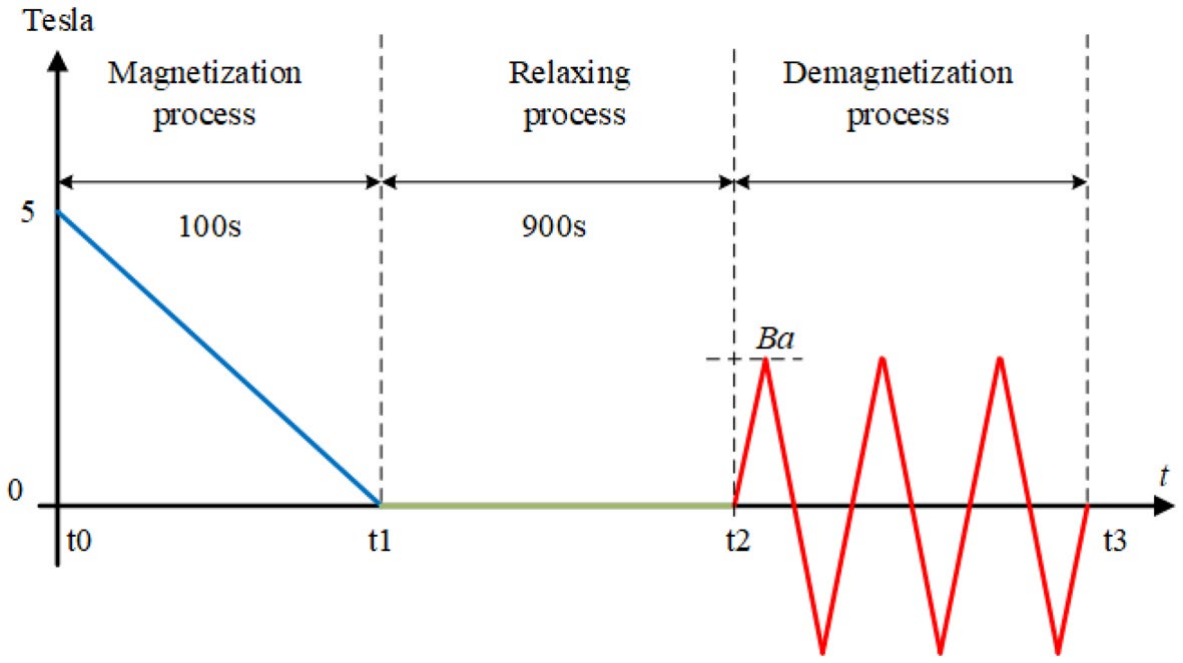
Sketch for the magnetization and demagnetization process. From t0 to t1, there is magnetizing applied magnetic field ($B_{app}$ in Fig. 1), which is perpendicular to the tape surface. From t1 to t2 there is no magnetic field, and from t2 to t3 the applied magnetic field is parallel and oscillating (demagnetizing cross-field, $B_{dem}$ in Fig. 1).

Here we assume that the magnetization and demagnetization processes are similar to past experiments, as follows^36,37^. The stack is initially magnetized along *y* axis, as defined in Fig. 1, by a field cooling process for 100 seconds under 5 T perpendicular direct magnetic field. This field is large enough to ensure the stack is completely magnetic saturation. And then, the stack is relaxed for 900 seconds. Subsequently, an alternating cross magnetic field with a triangular waveform is applied $B_{dem}$ to the stack (see Figs. 1 and 2). This process is the same as our previous work in^39^. The trapped field $B_{trapped}$ (along *y* axial direction) is observed at a distance of 1 mm above the top center surface of the stack. The trapped field of the partially coupled stack with a resistance in series is computed with standard mesh as 8 elements in thickness and 16 elements in width directions. Testing calculations were performed with varying the number of elements and the number of time steps in the cycle to verify the correct mesh strategy. This mesh strategy is precise enough and well balances the accuracy and computation time. The entire magnetization and demagnetization process are sketched in Fig. 2.

## Results and discussion

The electromagnetic properties of the stack are solved by the minimum electromagnetic entropy production method programmed in C++^40^. According to the slab critical state model (CSM)^49,50^, the parallel penetration field of the tape is $B_{p,tape}=\mu _{0} J_{c} d / 2 = 58.2$ mT for $d=1.5$ μm and $J_{c}=6.17 \times 10^{10}$ $\mathrm A/m^2$ ($I_c=3702.4$ A). Since there are 5 tapes in the stack, the penetration field of the stack in the full coupling limit (zero resistance at the ends) is 5 times of $B_{p,tape}$, which is $B_{p,stack}= 291$ mT.

For the computations, we assume a frequency of 2400 Hz, which is expected to be of the order of magnitude of the harmonics experienced in rotor stacks in motors for aviation, such as in^24^. Since the currents between tapes are of coupling nature^51^, they decrease with the average end resistance, $R_{av}$, but they increase with frequency. The reason is that the voltage in each coupling loop is proportional to the frequency. Then, the effect of duplicating the frequency will be roughly the same as dividing the end resistances by 2.

### The trapped field of the fully coupled stack and the uncoupled stack

**Figure 3:**
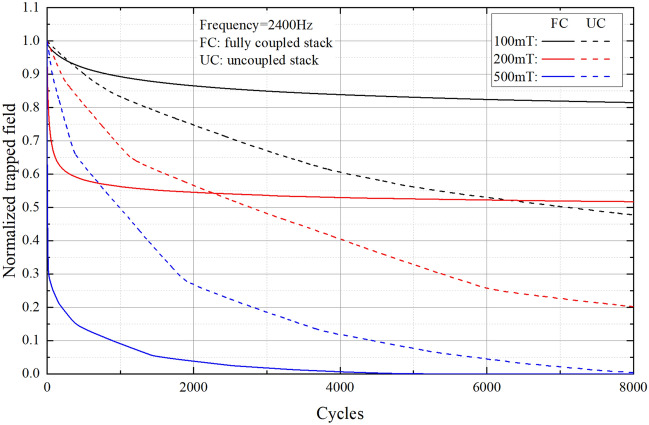
Normalized trapped field of the fully coupled stack and the uncoupled stack with a serious of cross-field amplitudes at frequency 2400 Hz. The solid lines are the fully coupled stack and the dash lines are the uncoupled stack, respectively.

Figure 3 shows the normalized trapped field of the fully coupled stack and the uncoupled stack with a series of cross-field amplitudes at the 2400 Hz frequency. Three amplitudes of 100, 200 and 500 mT respectively are chosen to study the trapped field.

For the uncoupled stack, the trapped field decays to $97.82,\;94.82$ and $87.84\%$ after 100 cross-field cycles with amplitudes 100, 200 and 500 mT respectively. Then, the trapped field continues to decay very fast even after thousands of cycles, which decay to $47.74,\;20.19$ and $0.39\%$ respectively after 8000 cycles. All these amplitudes are above the penetration field of a single tape (58.2 mT), which is the relevant quantity for complete demagnetization. For 100 and 200 mT cases, it simply takes more cycles to decay to zero. For example, a stack with 10 tapes could take hundreds of thousands of cycles to completely demagnetize^39^.

For the fully coupled stack (the resistance between tapes is zero), the decay is faster than the uncoupled stack (the resistance between tapes is infinitely large) at the first hundreds of cycles cross-field, which decay to $96.55,\;65.72$, and $24.13\%$, respectively. However, after one thousand cycles, the decay rate tends to be flat. After 8000 cycles, the trapped field still keeps $81.49$ and $51.72\%$ under cross-field with amplitude of 100 mT and 200 mT respectively, which is much higher than the uncoupled stack. This indicates that the fully coupled stack can trap a higher field than the uncoupled stack under the same cross-field.

Therefore, soldering with a sufficiently low resistance could greatly improve the cross-field demagnetization performance of the stacks.

### Trapped field of the partially coupled stack with resistances at terminals

**Figure 4:**
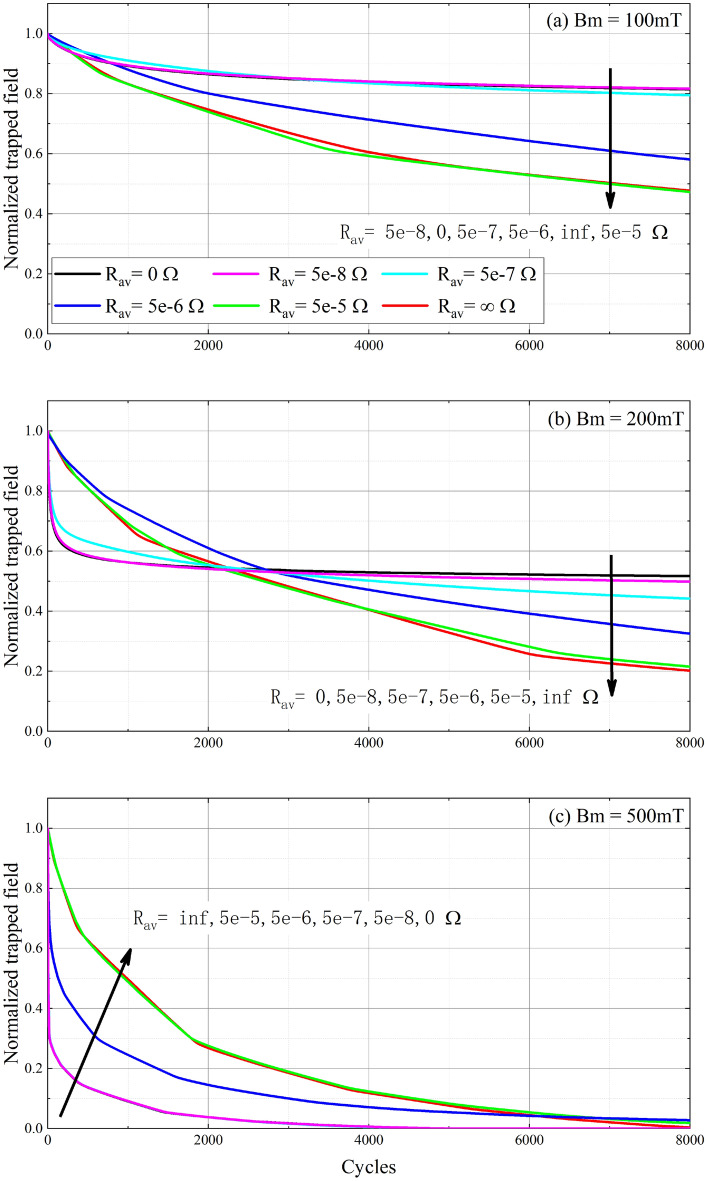
Normalized trapped field of three kinds of stack with a series of cross-field amplitudes at frequency 2400Hz . (**a**) $B_m =100$ mT, (**b**) $B_m=200$mT, and (**c**) $B_m=500$ mT. The black solid line is the fully coupled stack, the red solid line is the uncoupled stack, and the other color dash lines are the partially coupled stack with various average resistances.

Figure 4 illustrates the normalized trapped field of three types of stacks with a series of cross-field amplitudes at frequency 2400 Hz. The average resistances between tapes rang from $5\times 10^{-8}$ to $5\times 10^{-5}$ $\Omega$. It is clear that the trapped field is not only dependent on the amplitude of the cross-field, but also depends on the resistance value. When the resistance is minimal (such as $5\times 10^{-8}$ $\Omega$), the trapped field of the partially coupled stack is very close to the fully coupled stack. As the resistance increases, the trapped field changes towards the uncoupled stack case. When the resistance is large enough (such as $5\times 10^{-5}$ $\Omega$), the trapped field is the same as the uncoupled stack. This phenomenon is more obvious after thousands of cycles cross-field, especially at 200 mT, as shown in Fig. 4b.

### Current density in the partially coupled stack

**Figure 5:**
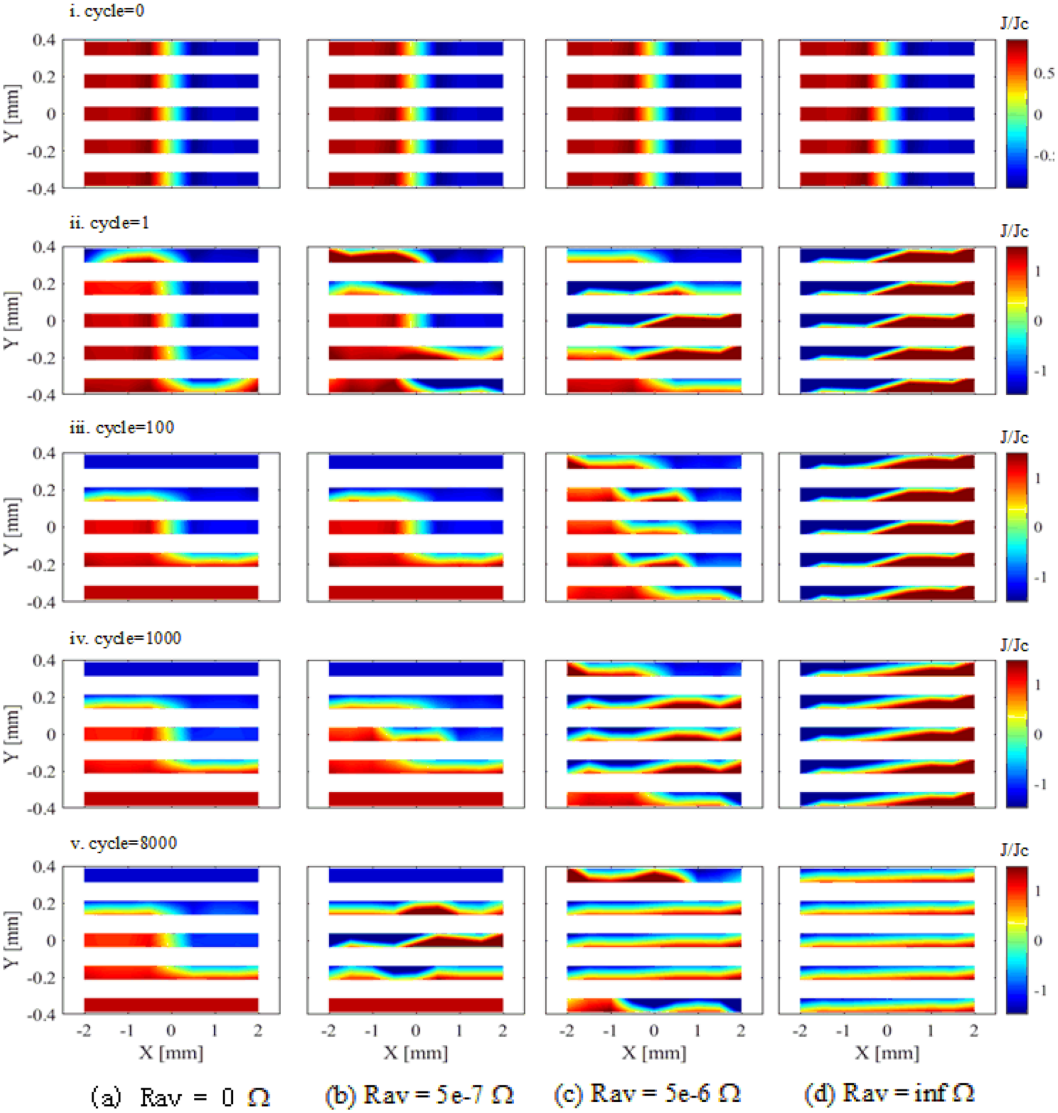
Current density maps of the stack suffering from 200 mT cross-field at frequency 2400 Hz. Five typical cycles are chosen, they are $cycle = 0,\;1,\;100,\;1000,$ and 8000. The thickness of the tapes are scaled 50 times to draw the pictures. (**a**) is the fully coupled stack which is the same as zero average end resistance, $R_{av} = 0$ $\Omega$. (**b**) and (**c**) are partially coupled stacks with average end resistance of $5\times 10^{-7}$ and $5\times 10^{-6}$ $\Omega$, respectively, and (**d**) is the uncoupled stack, where $R_{av} =\infty$ $\Omega$.

Figure 5 shows the current density maps of the stack with various resistances at terminals. When the resistance is zero (Fig. 5a), the current density among tapes presents as an S-shape, which is the same as a superconductor bulk after one thousand cycles cross-field. The current density map does not change much because the current can flow freely among the tapes. Subsequently, after thousands of cross-field cycles, the trapped field tends to be time independent.

As the resistance increases from $5\times 10^{-8}$ to $5 \times 10^{-5} \Omega$ (Fig. 5b–d), the current density map changes from the S-shape in the entire stack cross-section to inducted independently in each tape.

In addition, when the resistance increases from $5 \times 10^{-8} \Omega$, the power loss at the resistances, $P_R= \sum _{j}^{n_{tape}-1} R_{av} I_{\textrm{ta2ta},j}^2$, increases with the resistance,where $I_{ta2ta}$ is the tape-to-tape current. For sufficiently large resistances,however,the coupling currents in the loops decrease, and consequently the resistive AC loss decreases with the resistance. Therefore, the resistive AC loss presents a peak at a certain intermediate resistance. The results of the magnetization loss of the stack are provided in the the supplementary material.

When the resistance is large enough, the tapes are insulated from each other, and the currents will be inducted only within tapes, without tape-to-tape current at all (Fig. 5d). Additionally, the amplitude of the current density in each tape is smaller and smaller after thousands of cycles cross-field, so that the trapped field of the uncoupled stack always decays at a very slow rate.

In short, when a cross-field is applied, the tape-to-tape currents are inducted and the current density distribution in the tapes is affected accordingly. Consequently, the trapped field of the partially coupled stack must be located between the fully coupled stack and the uncoupled stack. To keep a stationary value, the ripple field amplitudes should be below the single tape parallel penetration field, of around 58.2 mT in our case. With the help of the resistance dependence, it is possible to design a stack with specific trap-field capabilities between the fully coupled stack and the uncoupled stack by adjusting resistance values.

### Tape-to-tape currents

**Figure 6:**
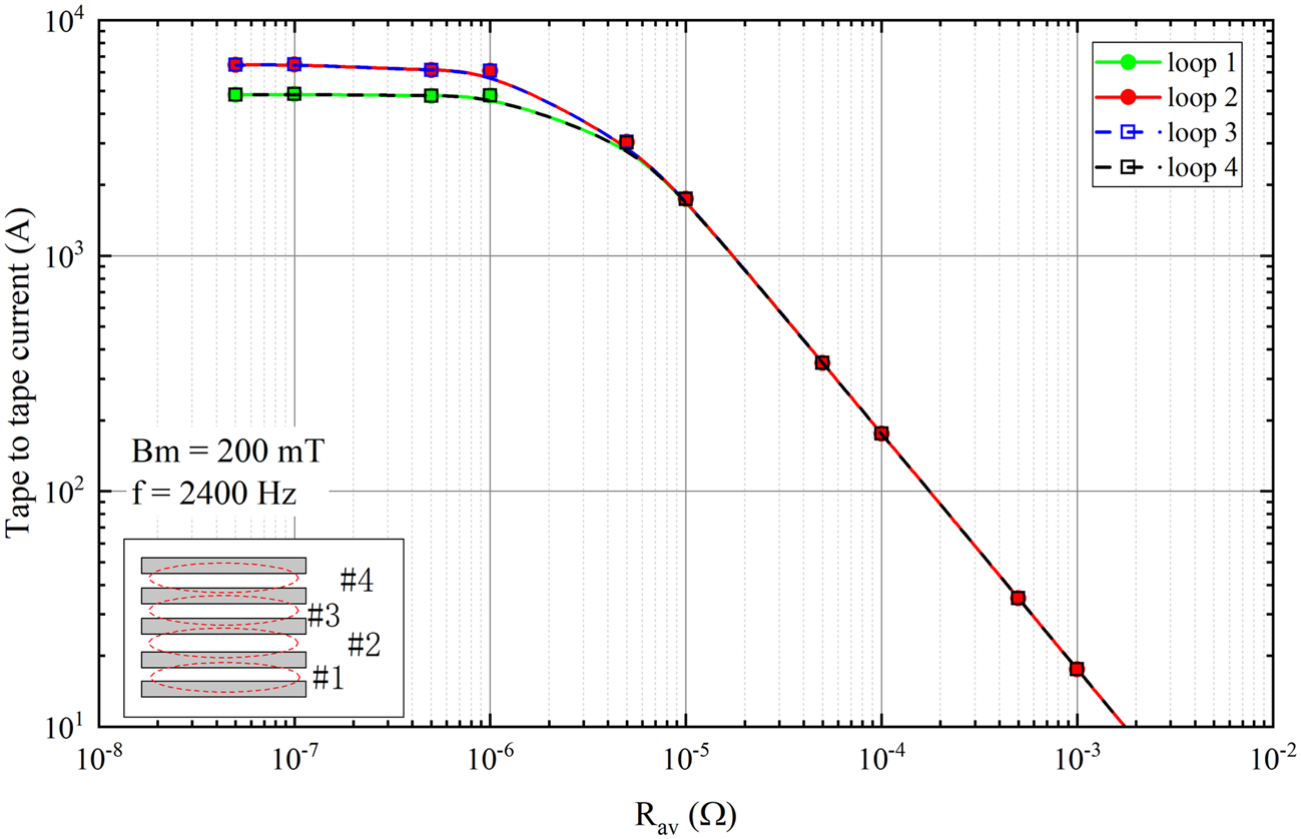
The tape-to-tape current amplitude when the stack is suffering from 200 mT cross-field. The resistance is increased from $5 \times 10^{-8}$ $\Omega$ to $1 \times 10^{-3}$ $\Omega$. Five tapes form four loops which are indexed from bottom to top in the sketch as 1 to 4.

Figure 6 illustrates the amplitudes of the tape-to-tape currents with various resistances. Five tapes form four loops indexed as 1 to 4 from bottom to top. Since there are resistances at terminals that form closed loop circuits, the tape-to-tape currents are inducted when the cross-field is applied. When the resistance is minimal, the tape-to-tape currents are almost constant. This is because the stack with low resistance performs more like a HTS bulk. As the resistance increases, the tape-to-tape currents begin to decrease, and this decrease is inversely proportional to the resistance for high enough resistances. This decrease in tape-to-tape current is logical, as the case of high resistance must approach to the uncoupled configuration where the tape-to-tape current vanishes.

## Conclusion

Here we study the trapped field of the partially coupled stack with 5 tapes and average resistances ranging from $5\times 10^{-8}$ to $5 \times 10^{-5} \Omega$ by the minimum electromagnetic entropy production method in 2D.

When a cross-field is applied on the partially coupled stack with resistances at terminals, the currents are inducted in superconducting tapes and resistances respectively. The resistances can enable tape-to-tape current under the parallel alternating magnetic field and then affect the current density distribution in the tapes. This has an impact on the trapped magnetic field and magnetization loss of the stacks.

On the contrary, for the uncoupled stack, which $R_{av}$ is infinity large, the tapes are insulated from each other and there is no tape-to-tape current either. The currents are only induced inside of each tape. Although the amplitude of the cross-field is the same as the fully coupled stack, the trapped field of the uncoupled stack always decays towards lower values, because the penetration field of the uncoupled stack is the same as a single tape, which is much smaller than the fully coupled stack.

The results indicate that the trapped field decay dependence of the stacks under cross-field are very different from each coupling configuration. For the fully coupled stack, where $R_{av}$ is zero, the trapped field decays very fast at the beginning but then it slows down and it tends to flatten. The reason is that when the resistance is zero, the stack behaves as an equivalent bulk with a parallel penetration field about five times higher. Then, soldered stacks can still keep permanent magnetization for relatively large AC parallel fields that fully demagnetize isolated stacks.

For the partially coupled stack with the $R_{av}$ increasing from $5\times 10^{-8}$ to $5 \times 10^{-5}$ $\Omega$, the tape-to-tape current shows monotone decrease. Accordingly, the current density in the stack shows a transient process from the fully coupled stack to the uncoupled stack. Then, the trapped field of the partially coupled stack is located somewhere between the fully coupled stack and the uncoupled stack. Thus, the trapped field is highly related to the resistance value.

In addition, when the resistance is very small or zero, the magnetization loss needs hundreds of cross-field cycles to reach stability. The magnetization loss of the partially coupled stack shows resistance dependence, in which the peak loss occurs around $R_{av}= 5\times 10^{-6}$ $\Omega$ in our case.

With the help of the resistance dependence on the trapped field and magnetization loss of the partially coupled stack, it is possible to design a stack with specific trap-field capabilities between the fully coupled stack and the uncoupled stack with low magnetization loss by adjusting resistance values at terminals. This designed partially coupled stack has a high potential to be used as the trapped field magnet in high-performance HTS applications in the near future, such as the electric motor for aircraft propulsion.

## Methods

The computations in this article use the Minimum Electro-Magnetic Entropy Production (MEMEP) method^35^. This method calculates the time evolution of the current density at any position of a sample, $\textbf{J}(\textbf{r},t)$, in materials with non-linear resistivity, such as superconductors. Indeed, REBCO high-temperature superconductors present a power-law relation between the current density, $\textbf{J}$, and the electric field, $\textbf{E}$, as

3 $$\begin{aligned} \textbf{E}(\textbf{J})=\frac{E_c}{J_c}\left( \frac{|\textbf{J}|}{J_c} \right) ^{n-1}{} \textbf{J}, \end{aligned}$$

where *n*, $J_c$, $E_c$ are independent on $\textbf{J}$ and are named as “power-law exponent” (or “*n*-value)”, “critical current density”, and “critical electric field criterion”, respectively. Although MEMEP enables non-homogeneous and magnetic-field dependent *n* and $J_c$, here we assume constant *n*, $J_c$, $E_c$ for simplicity, with values $n=30$, $J_c=6.17\cdot 10^{10}$ A/m$^2$, $E_c=10^{-4}$ V/m. In addition, MEMEP can also model normal conductors, where $\textbf{E}(\textbf{J})=\rho \textbf{J}$ and $\rho$ is the resistivity.

MEMEP can solve the current density in any three dimensional (3D) body by minimizing a certain functional^35^. As explained in^40^, for long tapes connected at the ends by a normal metal, the problem can be restricted to modeling the current density a the tape cross-section far away from the ends, reducing to a mathematically 2D problem (MEMEP 2D). In addition, the current density in the superconductor follows the infinitely long direction, which we choose as the *z* axis ($\textbf{J}=J{\hat{\textbf{z}}}$). As a result, the general 3D functional can be simplified into^40^

4 $$\begin{aligned} F=l_s\int _{\Omega _{2D,s}}\left[ \frac{1}{2} \frac{\Delta A_J}{\Delta t} \Delta J + \frac{\Delta A_a}{\Delta t}\Delta J + U(J_0+\Delta J) \right] \textrm{d}^2\textbf{r} + \sum _{j=1}^{n_t-1}RI_{\textrm{coupling},j}^2 . \end{aligned}$$

In the integral above, $l_s$ is the superconductor length; $\Omega _{2D,s}$ is the cross-sectional region of the superconductor at $z=0$; $J_0$ is the current density at the previous time step; $\Delta J=J-J_0$; the dissipation factor *U* is defined as $U(J)=\int _0^J\textrm{d}J'E(J')$; $\Delta A_a$ is the local change of applied vector potential between two time steps; and $\Delta A_J=A_J-A_{J_0}$, where $A_J$ and $A_{J_0}$ are the vector potentials in Coulomb’s gauge generated by *J* and $J_0$, which for infinitely long conductors is

5 $$\begin{aligned} A_J(\textbf{r})=-\frac{\mu _0}{2\pi }\int _{\Omega _{2D,s}} J(\textbf{r}')\ln |\textbf{r}-\textbf{r}'|\textrm{d}^2\textbf{r} . \end{aligned}$$

The additional sum in (4) accounts for the coupling currents in the end resistances, of value *R*. The net current in tape *j*, $I_j$, is related to the coupling currents, $I_{\textrm{coupling},j}$, as

6 $$\begin{aligned} I_{j}= \left\{ \begin{array}{ll} -I_{\textrm{coupling},1} &{} \text {for}\, j=1 \\ I_{\textrm{coupling},j-1}-I_{\textrm{coupling},j} &{} \text {for}\, 1<j < n_t \\ I_{\textrm{coupling},n_t-1} &{} \text {for}\, j=n_t \end{array}\right. . \end{aligned}$$

The optimized algorithm to minimize the functional in (4), and thence find $J(\textbf{r},t)$ and the coupling currents, $I_{\textrm{coupling},j}$, is detailed in^33,40^.

The reader should note that there were a few typos in^40^. The functional had a missing $l_s$ before the integral and there were also mistakes in the equation relating $I_{\textrm{coupling},j}$ and $I_j$. However, the computations in^40^ internally used the proper relations, and hence the results are correct.

## Supplementary Information

Supplementary Information.

## Data Availability

The datasets generated during and/or analysed during the current study are available from the corresponding author on reasonable request.
