# Supplementary material for: Numerical modelling of soldered superconducting REBCO stacks of tapes suggests strong reduction in cross-field demagnetization
Source: Sci Rep. 2023 Jan 19;13:1087. doi: 10.1038/s41598-023-27996-4 (PMC9852465; doi:10.1038/s41598-023-27996-4)
Supplement: Supplementary file 1 — Supplementary Information. [file 41598_2023_27996_MOESM1_ESM.pdf]

Supplementary material to: "Numerical modelling of soldered superconducting REBCO stacks of tapes suggests strong reduction in cross-field demagnetization"

Shuo Li[1,2], Enric Pardo[1]*

[1] Institute of Electrical Engineering, Slovak academy of sciences, v.v.i, 84104 Bratislava, Slovakia.

[2] College of Information Science and Engineering, Northeastern University, 110819 Shenyang, China.

* Corresponding author: enric.pardo@savba.sk

January 9, 2023

Magnetization loss of the stack under demagnetization cross-field

In this section, the magnetization loss of the stack is studied. When a cross-field with a triangle waveform is applied on the stack, superconducting currents and tape-to-tape currents are inducted in superconducting tapes and terminal resistances, respectively. All these currents cause magnetization loss. Here, we distinguish between the superconductor loss and resistance loss, as occurring in the superconductor and the resistance joints, respectively.

$$\begin{aligned} P(t) &= P_s(t) + P_R(t) \\ &= l_s \sum_{i}^{N} J_i(t) E_i(t) s_i + 2 \sum_{j}^{n_{tape}-1} R I^2_{\text{coupling},j}(t), \end{aligned} \tag{1}$$

where the first and second terms are the superconductor loss and resistance power loss, respectively.

The loss Q over one cycle is obtained by the integration of (1).

$$\begin{aligned} Q &= Q_s + Q_R \\ &= \int_{mT}^{(m+1)T} P_s(t)\mathrm{d}t + \int_{mT}^{(m+1)T} P_R(t)\mathrm{d}t \end{aligned} \tag{2}$$

where T is the period and m is the number of periods having been computed. Again, the first and second terms in (2) are the superconductor and resistance loss per cycle, respectively.

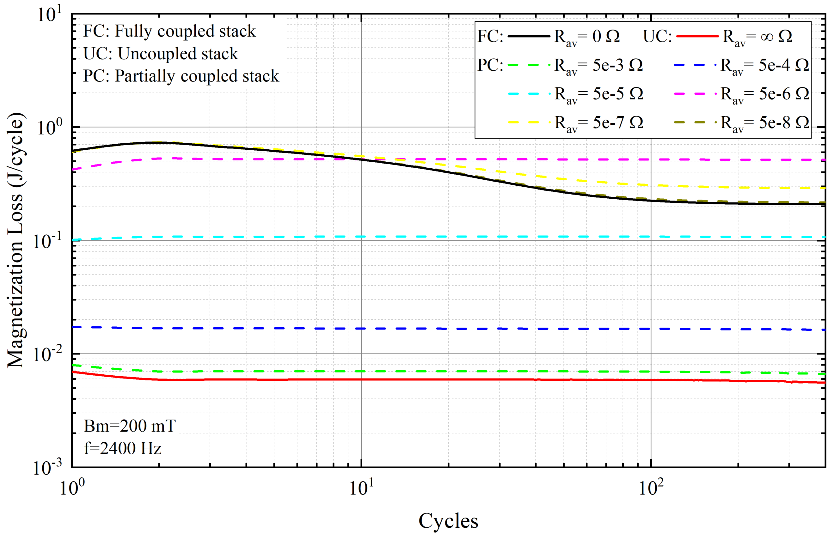

Figure 1: The magnetization loss of the stack with various resistances under an applied triangle waveform cross-field for 200 mT amplitude and 2400 Hz frequency. The black solid line is the fully coupled stack ($R_{av} = 0$), and the red solid line is the uncoupled stack ($R_{av} = \infty$). Other color dash lines are the partially coupled stack with resistance ranging from $5 \times 10^{-8}\Omega$ to $5 \times 10^{-3}\Omega$.

Fig. 1 shows the total magnetization loss of the stack at 200 mT 2400 Hz cross-field various resistances at terminals. It is easy to see, for the uncoupled stack ($R_{av} = \infty\ \Omega$), the magnetization loss reaches the stable value very soon, which takes only $2 \sim 3$ cycles. As the resistance decreases, the magnetization loss needs more and more cycles to achieve a stable state. When the resistance vanishes, the magnetization loss increases a little bit at the first few cycles and then decreases gradually in the coming one hundred cycles. This is because when the resistance is very small, the induced currents can flow between tapes, which need more cycles to stabilize the current density distribution (see Fig. 5 of the main manuscript). Once the current distribution is stable, the magnetization loss also becomes stable. Thus, the time required to stabilize the magnetization loss decreases with the resistance.

Fig. 2 shows the magnetization loss with a series of resistances. When the resistance is very small (close to $R_{av} = 1 \times 10^{-8}\ \Omega$), the resistor loss Q_R in the soldering parts is hundreds of times smaller than the superconducting loss Q_s in the superconductor tapes.

As the resistance increases up to $R_{av} = 1 \times 10^{-6}\ \Omega$, the tape-to-tape current

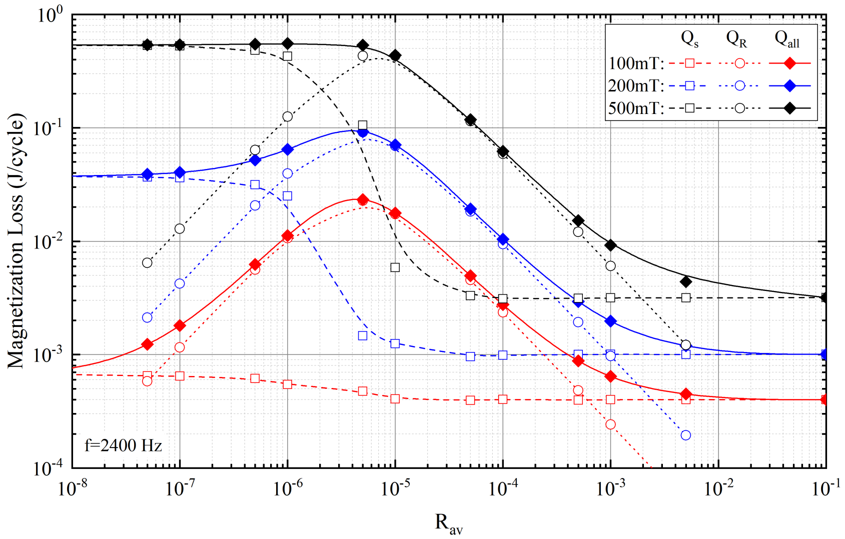

Figure 2: The magnetization losses of the stack with a series of resistances. The dash line with open rectangle symbols is the superconductor loss, the dot line with open circle symbols is the resistance loss, and the solid line with solid diamond symbols is the total loss. Here, $R = 1 \times 10^{-8}\ \Omega$ behaves is the same as fully coupled stack ($R = 0$) and $R = 0.1\ \Omega$ is the same as the uncoupled stack ($R = \infty$).

almost keeps constant (Fig. 6 of the main manuscript). Because the Joule loss in the resistance is directly proportional to the resistance value, the resistor loss Q_R increases linearly. When the resistance is around $R_{av} = 5 \times 10^{-6}\ \Omega$, the resistor loss Q_R reaches the peak, and the superconductor loss Q_s drops sharply. When the resistance further increases, there is no current in the soldered parts, and hence the Q_R decreases gradually towards zero. In the meantime, the superconductor loss Q_s keeps constant again.

At Last, the magnetization loss of the stack with large resistance is much lower than that with low resistance, and the magnetization loss of the partially coupled stack shows resistance dependence. The analytic formula for the resistance dependence of the magnetization loss was deduced in our previous work [1].

References

[1] Shuo Li, Ján Kováč, and Enric Pardo. Coupling loss at the end connections of REBCO stacks: 2D modelling and measurement. *Supercond. Sci. Technol.*, 33(7):075014, Jun 2020.
